# Salt-losing tubulopathy worsening the prognosis of renal sarcoidosis

**DOI:** 10.1007/s40620-022-01538-z

**Published:** 2023-01-16

**Authors:** Anis Abu Ayyach, Alain Le Moine, Louiza Kaci, Claire Royer-Chardon, Lidia Ghisdal, Martina Marangoni, Guillaume Smits, Joëlle Nortier

**Affiliations:** 1 Department of Nephrology, Dialysis and Transplantation, CUB Erasme, Université Libre de Bruxelles (ULB), Brussels, Belgium; 2 Human Pathology Laboratory, Algiers, Algeria; 3 Renal Pathology, CUB Erasme, ULB, Brussels, Belgium; 4 Department of Genetics, CUB Erasme, ULB, Brussels, Belgium; 5 Department of Nephrology-Dialysis, CHU Brugmann, Université Libre de Bruxelle (ULB), Place Van Gehuchten 4, 1020 Brussels, Belgium

**Keywords:** Systemic sarcoidosis, Renal sarcoidosis, Bartter-like syndrome, Salt-losing tubulopathy

## The case

**Fig. 1:**
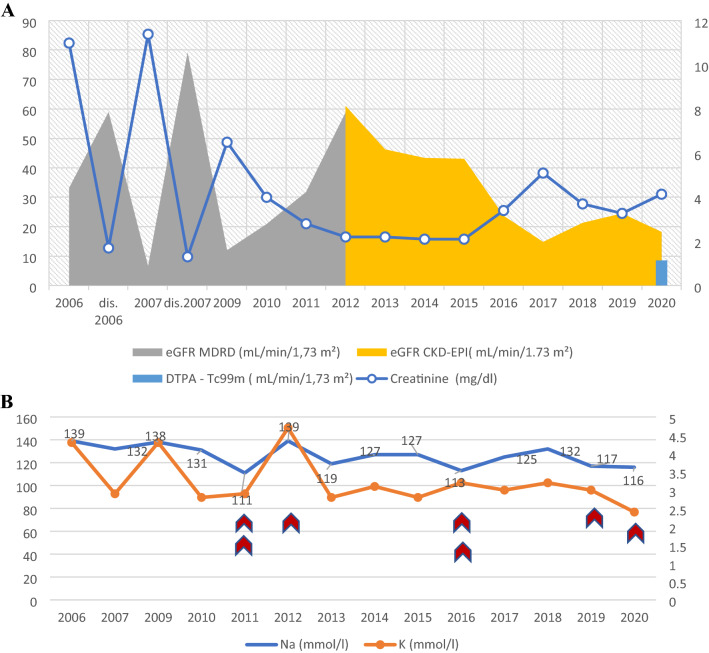
Time course of Scr, eGFR from 2006 to 2012 using the MDRD equation, and from 2012 to 2020 using the CKD-EPI equation; global GFR was determined by using the DTPA-Tc99m method (**A**); plasma levels of sodium (Na) and potassium (K) from 2006 to 2021 (**B**). Regular ICU admissions are noted during the clinical follow-up (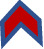)

We report the case of a 30-year-old north African male, the 7th child of consanguineous parents, initially treated in Algeria from 2006 to 2010. At the age of 14 years, he was admitted to hospital for fatigue, pallor and weight loss. Blood pressure was normal. Eye examination showed iridocyclitis. Blood tests confirmed hepatitis and acute kidney injury [AKI] with serum creatinine [Scr] of 11 mg/dl (Reference [ref] range 0.7–1.2 mg/dl) (Fig. 1). Considering a possible diagnosis of tubulointerstitial nephritis and uveitis (TINU) syndrome, high doses of local and i.v. methylprednisolone followed by oral doses were started, leading to a significant but transient improvement of kidney (Scr 1.7 mg/dl) and liver parameters.

One year later, the patient experienced recurrent iridocyclitis and AKI (Scr 11.4 mg/dl) accompanied by severe hypokalemia (2.9 mEq/l; ref. range 3.5–4.8), hyponatremia (132 mEq/l; ref. range 136–145), hypochloremia (80 mmol/l; ref. range 98–107) and metabolic alkalosis (36 mmol/l; ref. range 22–29), suggesting an acquired Bartter-like syndrome (Fig. 1B). Indomethacin administration and potassium supplementation were initiated.

**Fig. 2:**
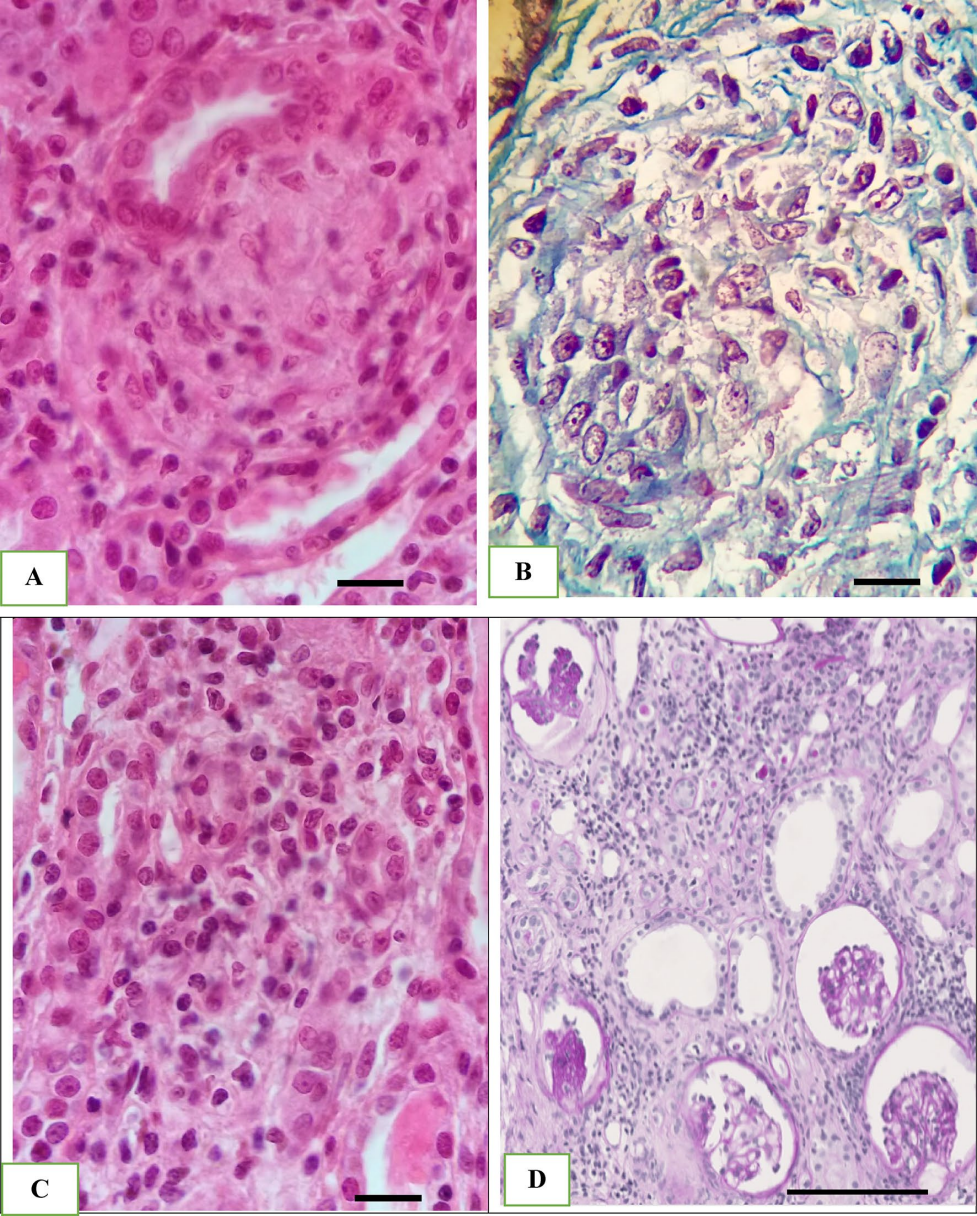
First kidney biopsy showing non caseating granuloma (HE staining, magnification, × 100) (**A**) epitheloid granuloma (Masson’s trichrome staining, magnification, × 100) (**B**) and mononuclear cell infiltration (HE staining, magnification, × 100) (**C**). Second kidney biopsy exhibiting extensive tubulo-interstitial nephritis lesions (PAS staining magnification × 12.8) (**D**). Image (A,B and C) (scale bar 20 µm), image (D) (scale bar 200 µm)

A first kidney biopsy showed noncaseating granulomas and tubulointerstitial infiltrates (Fig. 2A–C). Intravenous corticosteroids improved kidney function (nadir of Scr 1.3 mg/dl). The diagnosis of sarcoidosis had been suggested in 2009 on the basis of a very high level of angiotensin converting enzyme (193 U/L; ref. range 12–68).

In 2010, the patient was admitted to our department. Indomethacin was discontinued due to hepatitis and renal failure. Hypokalemia, hypercalcemia and hyponatremia were treated by replacement of fluids and electrolytes. Pituitary disorders and primary hyperaldosteronism were ruled out by hormonal tests; autoimmune tests were negative. Oral corticosteroids (prednisolone 16 mg/day) and potassium supplements were maintained. A liver biopsy and a second kidney biopsy were performed. Extensive tubulointerstitial nephritis and a liver granuloma were detected, confirming the diagnosis of systemic sarcoidosis (Supplemental material).

In 2017, a third episode of AKI (Scr increased to 5 mg/dl) with hypercalcemia (2.93 mmol/l) and a very high level of 1.25 dihydroxy-vitamin D (268 ng/l) occurred, suggesting sarcoidosis relapse. No sign of nephrocalcinosis was detected at imaging.

Over the last 10 years, seven intensive care unit (ICU) admissions for severe hyponatremia with life-threatening neurological complications, including seizures, confusion and behavior disorders, were required (Fig. 1B). Extensive neurological tests were performed, and neurosarcoidosis was ruled out.

However, chronic kidney disease worsened, leading to hemodialysis initiation in September 2020.

## Lessons for the clinical nephrologist

Sarcoidosis is a multisystem granulomatous disease of unknown origin; it may be triggered by environmental factors (such as infection, drug toxicity) in individuals with a genetic predisposition [1, 2]. Sarcoidosis may affect any organ, but mostly involves lungs and lymph nodes; besides the typical histological finding of noncaseating granulomatous interstitial nephritis, kidney manifestations are uncommon and may be underreported, and can include calcium homeostasis disorders, glomerular disease, tubular dysfunction, obstructive and vascular uropathy [1–4].

The present case illustrates how the diagnosis of systemic sarcoidosis with renal involvement is often delayed. The first diagnostic hypothesis was TINU syndrome because of ocular findings (granulomatous iridocyclitis) and interstitial nephritis at the first kidney biopsy. Differential diagnosis considered Sjögren’s syndrome, tuberculosis, systemic lupus erythematosus, granulomatosis with polyangiitis and Behçet’s syndrome.

Corticosteroids remain the cornerstone for the treatment of tubulointerstitial granulomatosis in renal sarcoidosis [1, 8]. Our patient’s clinical situation worsened in spite of long-term corticosteroid administration. Moreover, corticosteroid therapy was responsible for acquired Cushing syndrome, spinal deformations, and amyotrophy (Fig. 2b). In fact, severe osteoporosis was already present in 2010 (T-score − 4.9 at the level of the lumbar spine and − 2.9 at the level of the femoral neck), and had significantly worsened by 2018 (respective T-scores − 6.2 and − 3.2). In such a setting, other therapeutic strategies such as azathioprine or mycophenolate mofetil could have been considered [1].

The association of sarcoidosis with salt-losing nephropathy is a life-threatening condition related to ionic disturbances. The alternative diagnoses of Bartter and Gitelman syndrome should be considered, as these genetic conditions characterized by tubulopathy affecting the thick ascending limb of the loop of Henle, lead to ionic disorders (hypokalemia, hypocalcemia with hypercalciuria) and are associated with metabolic alkalosis, hyperreninemic hyperaldosteronism and normal blood pressure parameters [5]. In our case, blood pressure was normal. Blood tests revealed hypokalemia, hyponatremia, hypochloremia with metabolic alkalosis HCO3 at 33 mmol/l, confirmed on venous blood gas (pH 7.47). Blood magnesium was normal, however, severe hypercalcemia (11 mg/dl) was found and plasma renin and aldosterone levels were increased: renin 4600 ng/ml (ref. range 0.84–2.5) and aldosterone 3903 pg/mL (ref. range 5.7–240), respectively. Potassium wasting was significant (298 mmol/l/24 h, ref. range 25–125) as was natriuresis wasting (479 mmol/l/24 h, ref. range 40–220). Hyper calciuria was absent upon admission and during our follow-up. Cases of pseudo Bartter/Bartter-like syndrome have been reported without association with hypercalciuria [9].

To our knowledge, this is the second reported case of acquired Bartter syndrome with renal involvement in systemic sarcoidosis.

Barter-like syndrome (pseudo Bartter) can be inherited or acquired; the inherited syndrome can be divided into five subtypes: types I–IV are due to loss-of-function mutations in the Na/K/Cl co-transporter (NKCC2), ATP-regulated potassium channel (ROMK), kidney-specific basolateral chloride channel (CLCNKB) and *β*-subunit of the basolateral chloride channel (BSND), while type V is due to gain-of-function mutations in calcium-sensing receptors (CASR) [6]. Acquired Bartter syndrome is commonly associated with tuberculosis, autoimmune disease (Sjogren’s syndrome), sarcoidosis, diuretic abuse, or drugs such as aminoglycosides, tuberculostatics (capreomycin and viomycin), amphotericin B and cisplatinum [6, 7].

Considering the presence of consanguinity in our patient’s family and the suspicion of pseudo Bartter syndrome, an extensive genetic analysis was performed. No abnormality of the following genes was found: SLC12A3 (Gitelman syndrome), SLC12A1 (Bartter type 1), KCNJ1 (Bartter type 2), CLCNKB (Bartter type 3), BSND (Bartter type 4a), CLCNKA (Bartter type 4b), CASR (Bartter type 5).

Treatment with potassium supplementation, spironolactone and indomethacin was initiated early in our patient. Indomethacin (like other NSAIDs) is frequently given to treat Pseudo Barrter/Bartter-like syndrome [8]. Recurrent hepatitis and deleterious side effects of indomethacin on kidney function are however well known limiting factors for its long-term use [8].

## Supplementary Information

Below is the link to the electronic supplementary material.

Supplementary file1 (DOCX 923 kb)

## References

[1] Hilderson I, Van Laecke S, Wauters A, Donck J (2014). Treatment of renal sarcoidosis: is there a guideline? Overview of the different treatment options. Nephrol Dial Transplant.

[2] Stehléa T, Boffab J-J, Langa P, Desvauxc D, Sahalia D, Audarda V (2012) Atteintes rénales de la sarcoïdose. Rev Med Interne; pii: S0248–8663(12)00722-9 10.1016/j.revmed.2012.10.009 23154110

[3] Bergner R, Löffler C (2018). Renal sarcoidosis: approach to diagnosis and management. Curr Opin Pulm Med.

[4] Correia FASC, Marchini GS, Torricelli FC, Danilovic A, Vicentini FC, Srougi M, Nahas WC, Mazzucchi E (2020). Renal manifestations of sarcoidosis: from accurate diagnosis to specific treatment. Int Braz J Urol.

[5] Bartter FC, Pronove P, Gill JR, MacCardle RC (1962). Hyperplasia of the juxtaglomerular complex with hyperaldosteronism and hypokalemic alkalosis. A new syndrome. Am J Med.

[6] Tung-Min Yu, Lin S-H, Ya-Wen C, Wen M-C, Chen Y-H, Cheng CH, Chen C-H, Chung-Shi C, Shu K-H (2009). A syndrome resembling Bartter’s syndrome in sarcoidosis. Nephrol Dial Transplant.

[7] Dalugama C, Pathirage M, Kularatne SAM (2018). Bartter syndromelike phenotype in a patient with diabetes: a case report. J Med Case Reports.

[8] Lesley R, Paul B, Detlef B, Nicholas W (2012). Pediatric nephrology.

[9] Matsunoshita N (2015). Differential diagnosis of Bartter syndrome, Gitelman syndrome, and pseudo–Bartter/Gitelman syndrome based on clinical characteristics.

