# Supplementary material for: Salt-losing tubulopathy worsening the prognosis of renal sarcoidosis
Source: J Nephrol. 2023 Jan 16;36(3):627–30. doi: 10.1007/s40620-022-01538-z (PMC10089998; doi:10.1007/s40620-022-01538-z)
Supplement: Supplementary file 1 — Supplementary file1 (DOCX 923 kb) [file 40620_2022_1538_MOESM1_ESM.docx]

**Supplement material**

| **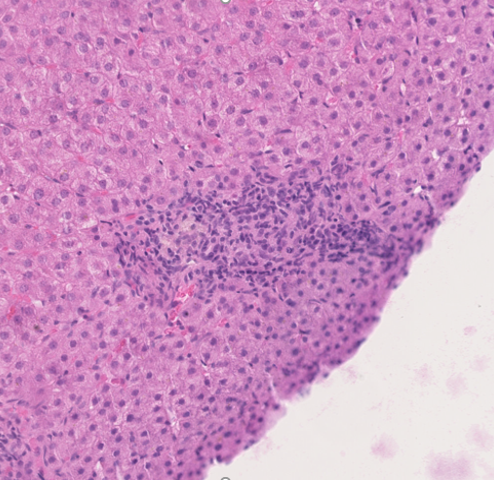**  **Figure 1b.** Unilobular granuloma detected in the liver biopsy (HE staining, magnification x20)( (scale bar 100 µm) |  |
| --- | --- |


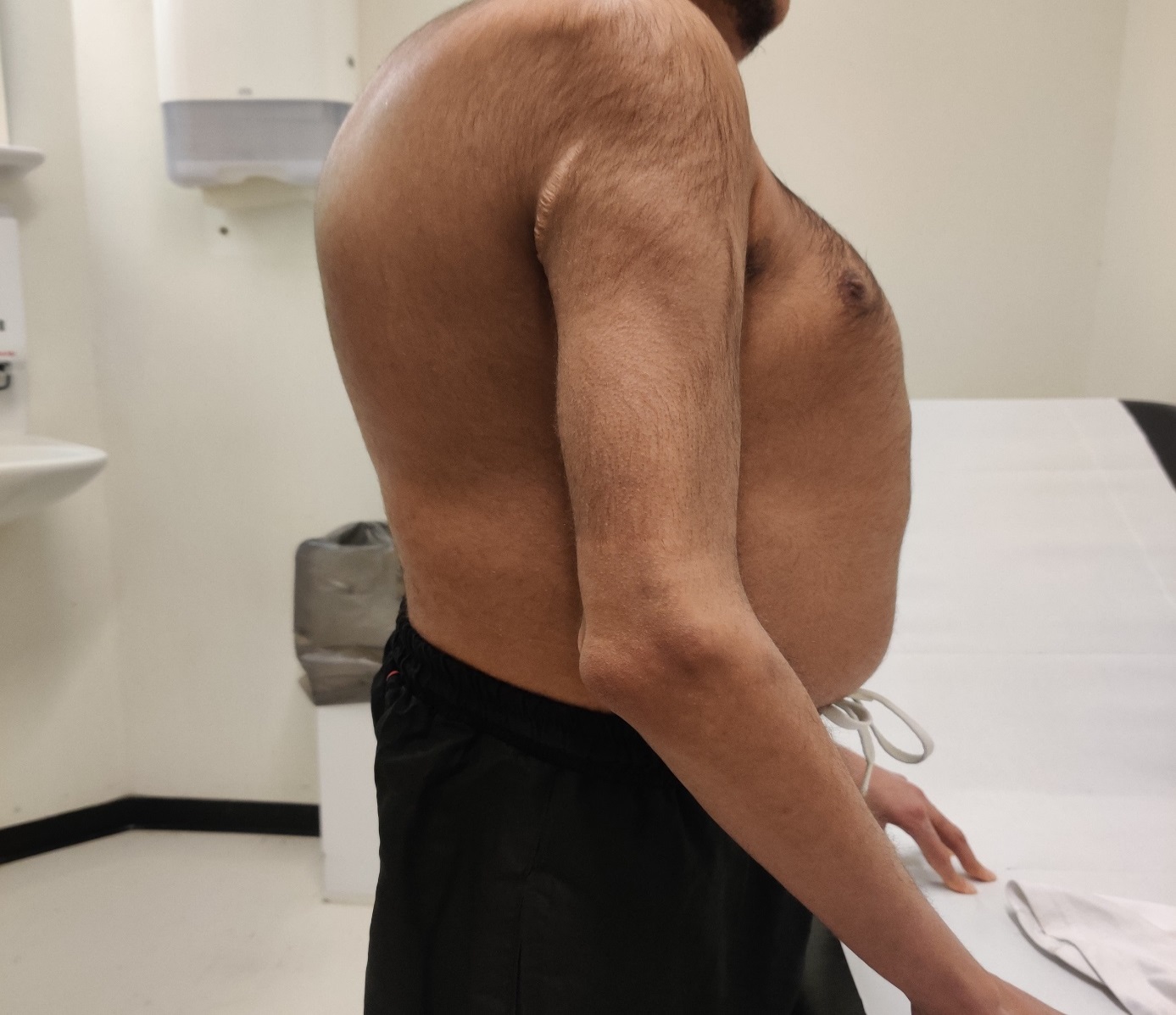


**Figure 2b**. Typical hunchback and stretch marks observed after long term corticosteroids treatment in this patient.
